# Severe malocclusion and oral health-related quality of life in adolescents aged 12-15 years

**DOI:** 10.1590/2177-6709.29.6.e2424100.oar

**Published:** 2025-01-13

**Authors:** Cibele da Cruz Prates OLIVEIRA, Marijara Vieira de Sousa OLIVEIRA, Carlos Antônio Amaro LIRA, Niely Enetice de Sousa CATÃO, Luana de Carvalho LOURENÇO, Rebecca Durand Garrido RAMALHO, Alessandro Leite CAVALCANTI, Alidianne Fábia Cabral CAVALCANTI

**Affiliations:** 1 Paraíba State University, Dentistry Program (Campina Grande/PB, Brazil)

**Keywords:** Malocclusion, Socioeconomic factors, Quality of life, Má oclusão, Fatores socioeconômicos, Qualidade de vida

## Abstract

**Introduction:**

Malocclusion is a public health problem. The evidence of its impact on quality of life is contradictory and requires further studies.

**Objective:**

The aim of this study was to estimate the prevalence of severe malocclusion and its impact on oral health-related quality of life in schoolchildren aged 12-15 years.

**Methods:**

A cross-sectional study was conducted with a probabilistic sample of 391 students. A sociodemographic questionnaire was used to collect information regarding the family context. The presence of malocclusion was assessed using the Dental Aesthetic Index (DAI), and oral health-related quality of life (OHRQoL) was analyzed using the Oral Health Impact Profile (OHIP). A descriptive and bivariate analysis of data was carried out. Multivariate Poisson regression models were used. The significance level adopted was 5%.

**Results:**

The prevalence of severe malocclusion was 37.6%, being 8.4% higher in the group aged 12-13 years (95%CI=1.48-2.29; p=0.034); and 11.2% higher (95%CI %=1.43-2.06; p=0.020) among those who perceived that their teeth were poorly positioned. Severe malocclusion was not associated with OHRQoL (p=0.686).

**Conclusions:**

The psychological discomfort, social disability and psychological disability domains had a negative impact on OHRQoL. However, there was no negative impact of severe malocclusion on OHRQoL.

## RESUMO

**Introdução:**

A má oclusão é um agravo que constitui um problema de saúde pública. As evidências do seu impacto na qualidade de vida são contraditórias e necessitam de outros estudos.

**Objetivo:**

O objetivo deste estudo foi estimar a prevalência de má oclusão severa e o seu impacto na qualidade de vida relacionada à saúde bucal em escolares de 12 a 15 anos de idade.

**Método:**

Foi conduzido um estudo transversal com uma amostra probabilística de 391 estudantes. Um questionário sociodemográfico foi utilizado para reunir informações relativas ao contexto familiar. A presença de má oclusão foi avaliada pelo *Dental Aesthetic Index* (DAI) e a qualidade de vida relacionada à saúde bucal (QVRSB) foi analisada pelo *Oral Health Impact Profile* (OHIP). Realizou-se uma análise descritiva e bivariada dos dados. Modelos multivariados de regressão de Poisson foram empregados. O nível de significância adotado foi de 5%.

**Resultados:**

A prevalência de má oclusão severa foi de 37,6%, sendo 8,4% maior na faixa etária de 12 a 13 anos (IC 95% = 1,48-2,29; p=0,034) e 11,2% maior (IC 95%=1,43-2,06; p=0,020) entre aqueles que perceberam possuir dentes mal posicionados. A má oclusão severa não se mostrou associada à QVRSB (p=0,686).

**Conclusão:**

Os domínios desconforto psicológico, incapacidade social e incapacidade psicológica tiveram impacto negativo na QVRSB. No entanto, não houve impacto negativo da má oclusão grave na QVRSB.

## INTRODUCTION

Malocclusions are considered a public health problem on a global scale, justified by their wide prevalence and the negative impacts on people’s lives.^1^ This problem affects function, appearance, social life and self-esteem of individuals, and this set of items are important constructs related to quality of life,^2^ especially in the group of adolescents, as the importance of their colleagues’ opinion about their oral health condition is evident. Individuals with malocclusion may be bullied more frequently.^3^ ^,^ ^4^

The severity of malocclusion has generally been investigated using occlusal indices and, in this context, the Dental Aesthetic Index (DAI)^5^ has stood out, which since its formulation has been commonly used in epidemiological studies in countries such as Brazil^6^ and Finland.^7^

It has been highlighted in literature that the greater the degree of severity, the greater the repercussions.^8^ ^,^ ^9^ Therefore, the occurrence of severe malocclusion has been related to higher probability of compromising quality of life, considering that in addition to the impact on aesthetics, it generates difficulties in hygiene, chewing and swallowing, as well as in the way of articulating and pronouncing words.^10^ ^,^ ^11^

A recent systematic review showed that malocclusions in adolescents have a negative impact on oral health-related quality of life (OHRQoL), after taking relevant confounders, such as age, gender, caries, and socioeconomic status, into consideration. Therefore, given these findings, it is important to establish whether this condition really has a negative impact on health-related quality of life (HRQoL) in this population group.^12^

In view of the above, assuming that HRQoL is influenced by severe malocclusion, this study aimed to identify the prevalence of severe malocclusion and evaluate the impact of this problem on the OHRQoL of schoolchildren aged 12-15 years.

## MATERIAL AND METHODS

This cross-sectional study was carried out in accordance with the Strengthening the Reporting of Observational Studies in Epidemiology (STROBE) checklist guidelines for observational studies.^13^ The research was conducted in a medium-sized municipality (HDI = 0.72 and Gini Index = 0.58) in the state of Paraíba, located in Northeastern Brazil, between March and July 2023.

The probabilistic cluster type sample was composed of 391 students and calculated using the OpenEpi digital tool (www.OpenEpi.com), considering the infinite population formula, confidence level of 95%, prevalence of severe malocclusion of 30.4%^13^ and design effect of 1.2.

**Figure 1:**
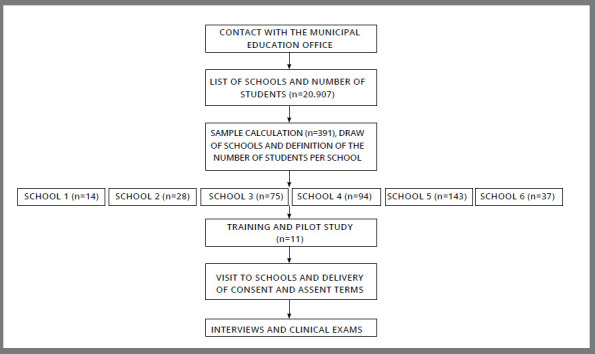
Study flowchart.

The selection of adolescents took place in two stages: in the first stage, six public schools in the municipal education network were drawn; and in the second stage, students were randomly chosen (Fig 1). Regularly enrolled students of both sexes aged 12-15 years, with complete permanent dentition and who did not use orthodontic appliances were included. Students who had already undergone previous orthopedic or orthodontic treatment, as well as non-cooperative students and syndromic students whose intraoral physical examination was unfeasible were excluded from the sample.

Variables related to the student were gender (male and female), age group (12-13 years old and 14-15 years old), ethnicity (white and non-white), visit to the dentist (yes or no), time from the last dental visit (≤ 6 months and > 6 months) and self-perception of poorly positioned teeth (yes or no). With regard to the family context, type of arrangement was researched (nuclear = couple with children; and non-nuclear = other configurations), maternal schooling (≤ 8 years of study and > 8 years of study), monthly income (up to 1 minimum wage and more than 1 minimum wage) and the granting of social benefits (yes or no). This information was collected through a questionnaire, which was subjected to a pre-test during the execution of the pilot study, which included the participation of 11 adolescents, whose data were not included in the main study.

To investigate malocclusion, classify its severity and need for orthodontic treatment, the Dental Aesthetic Index (DAI) was used.^5^ According to the final score obtained, four malocclusion categories were established: 1) normal occlusion/no need for treatment (≤ 25); 2) established malocclusion/need for elective treatment (26-30); severe malocclusion/need for highly desirable treatment (31-35); very severe malocclusion/need for mandatory treatment (≥ 36). Three calibrated examiners carried out the assessments, with inter- and intra-examiner agreements being calculated using the Cohen’s Kappa coefficient (κ ≥ 0.95).

For this study, the variable severe malocclusion was dichotomized into absent (DAI < 31) and present (DAI ≥ 31).^14^ As for DAI components, the number of missing teeth was dichotomized in “none” and “one or more”; median diastema, in “no” and “yes”; anterior misalignment (upper and lower), in “< 2mm” and “≥ 2mm”; overjet (maxillary and mandibular), in “< 4mm” and “≥ 4mm”; and anterior open bite, in “< 2mm” and “≥ 2mm”.^15^

The Oral Health Impact Profile (OHIP-14) was used to evaluate the impact of severe malocclusion on OHRQoL. This instrument, composed of 14 questions, presents seven domains: functional limitation, physical pain, psychological discomfort, physical disability, psychological disability, social disability and disability. The answer options for each of the questions are 0 = “never”; 1 = “a few times”; 2 = “sometimes”; 3 = “almost always”; 4 = “always”; and the total score ranges from 0 to 56. Higher values indicate negative impact on OHRQoL.^16^ For the OHRQoL evaluation, the median (OHIP-14 = 10.00) was adopted as the cutoff point for dichotomization, into “without impact” and “with impact”.^6^

Descriptive analysis of data was carried out (absolute and relative frequencies, for qualitative variables, measures of central tendency and variability, for quantitative variables) and then the Pearson Chi-square or Fisher’s Exact tests were used to identify possible associations between dependent variables (severe malocclusion and OHRQoL) and independent variables related to socioeconomic data and oral health of adolescents. Using Poisson Regression analysis with robust variance, crude and adjusted prevalence ratios were estimated, with their respective 95% confidence intervals. All variables with p<0.20 in the bivariate analysis were entered into the regression. Significance level of 5% was adopted, and the IBM SPSS software (version 24.0 for Windows, IBM Corp., Armonk, NY, USA) was chosen to construct the database.

The research was approved by the Research Ethics Committee (4,974,026) and national and international legislation related to ethics in research involving human beings was followed.

## RESULTS

Most participants were male (50.4%) and non-white (74.7%). The majority had history of visiting the dentist (75.7%) and the last visit took place in less than six months (60.8%). Furthermore, they reported the presence of poorly positioned teeth (65.2%) (Table 1).

**Table 1:** Distribution of adolescents according to age group, ethnicity, visit to the dentist, time of last dental appointment and self-perception of poorly positioned teeth, according to gender.

Variables	Gender					
	Male		Female		Total	
	n	%	n	%	n	%
Age group						
12-13 years old	128	47.6	141	52.4	269	100.0
14-15 years old	69	56.6	53	43.4	122	100.0
Ethnicity						
White	47	51.6	44	48.4	91	100.0
Non-white	148	50.7	144	49.3	292	100.0
Visit to the dentist						
Yes	147	49.7	149	50.3	296	100.0
No	50	52.6	45	47.4	95	100.0
Time from the last dental visit						
≤ 6 months	29	39.2	45	60.8	74	100.0
> 6 months	118	53.2	104	46.8	222	100.0
Self-perception of poorly positioned teeth						
Yes	23	34.8	43	65.2	66	100.0
No	174	53.7	150	46.3	324	100.0

Table 2 shows prevalence of non-nuclear family arrangement (59.0%), mothers who had eight or more years of schooling (58.0%), monthly family income of up to one minimum wage (61.9%) and families receiving social benefits (53.6%).

**Table 2:** Distribution of students according to variables related to family context.

Variables	n	%
Type of arrangement		
Nuclear	159	41.0
Non-nuclear	229	59.0
Maternal schooling		
≤ 8 years of study	163	42.0
> 8 years of study	225	58.0
Monthly income		
Up to 1 minimum wage ^+^	234	61.9
More than 1 minimum wage	144	38.1
Social benefits ^++^		
Yes	208	53.6
No	180	46.4

^+^ Brazilian minimum wage in force at the time of the research equivalent to US$ 264. ^++^Bolsa Família.

Maxillary overjet ≥ 4 mm was the most prevalent DAI component (67.8%), followed by dental crowding (64.4%). Spacing in the anterior segment was found in 41.7% of adolescents, while superior anterior misalignment ≥ 2 mm, in 23.8%. The prevalence of severe malocclusion was 37.6% (Table 3).

**Table 3:** Distribution of adolescents according to DAI components, presence of severe malocclusion and severity of malocclusion.

DAI components	n	%
Number of missing teeth		
None	376	96.2
One or more	15	3.8
Assessment of crowding in the incisal segments		
No segments crowded	139	35.5
1 segment crowded	122	31.2
2 segments crowded	130	33.2
Assessment of spacing in the incisal segments		
No segments spaced	228	58.3
1 segment spaced	110	28.1
2 segments spaced	53	13.6
Median diastema		
No	302	77.2
Yes	89	22.8
Anterior misalignment on the maxilla		
< 2 mm	298	76.2
≥ 2 mm	93	23.8
Anterior misalignment on the mandible		
< 2 mm	349	89.3
≥ 2 mm	42	10.7
Maxillary overjet		
< 4 mm	126	32.2
≥ 4 mm	265	67.8
Mandibular overjet		
< 4 mm	383	98.0
≥ 4 mm	8	2.0
Anterior open bite		
< 2 mm	377	96.4
≥ 2 mm	14	3.6
Antero-posterior molar relation		
Normal	117	29.9
1/2 cusp either mesial or distal	223	57.0
One full cusp or more either mesial or distal	51	13.0
Severe malocclusion		
Absent	244	62.4
Present	147	37.6
Malocclusion categories		
Normal occlusion	127	32.5
Established malocclusion	117	29.9
Severe malocclusion	70	17.9
Very severe malocclusion	77	19.7

The median OHIP-14 score was 10.00, with interquartile range (IIQ_25-75_) of 5.00-18.00. The domains with the highest frequency of negative impact on OHRQoL were psychological discomfort (34.0%), social disability (26.3%) and psychological disability (20.7%) (Table 4).

**Table 4:** Prevalence of negative impact on adolescents’ OHRQoL by OHIP-14 domain.

OHIP-14 Domain	Without impact	With impact
	n (%)	n (%)
1. Functional limitation	356 (91.0)	35 (9.0)
2. Physical pain	353 (90.3)	38 (9.7)
3. Psychological discomfort	258 (66.0)	133 (34.0)
4. Physical disability	365 (93.4)	26 (6.6)
5. Psychological disability	310 (79.3)	81 (20.7)
6. Social disability	288 (73.7)	103 (26.3)
7. Disability	342 (87.5)	49 (12.5)

Statistically significant associations were identified between severe malocclusion and age group (p=0.046), as well as with self-perception of poorly positioned teeth (p=0.024). Regarding OHRQoL, associations were observed with gender (p<0.001), self-perception of poorly positioned teeth (p<0.001) and type of family arrangement (p=0.013). However, OHRQoL was not affected by DAI components, nor by the presence of severe malocclusion or severity of malocclusion (Table 5).

**Table 5:** Association between dependent variables, presence of severe malocclusion and impact on adolescents’ OHRQoL, and independent variables.

Variables	Severe malocclusion							OHRQoL						
	Absent		Present		Total		p-value	Without impact		With impact		Total		p-value
	n	%	n	%	n	%		n	%	n	%	n	%	
Gender														
Male	124	62.9	73	37.1	197	100.0	0.824*	128	65.0	69	35.0	197	100.0	<0.001*
Female	120	61.9	74	38.1	194	100.0		69	35.6	125	64.4	194	100.0	
Age group														
12-13 years old	159	59.1	110	40.9	269	100.0	0.046*	132	49.1	137	50.9	269	100.0	0.441*
14-15 years old	85	69.7	37	30.3	122	100.0		65	53.3	57	46.7	122	100.0	
Ethnicity														
White	52	57.1	39	42.9	91	100.0	0.260*	45	49.5	46	50.5	91	100.0	0.882*
Non-white	186	63.7	106	36.3	292	100.0		147	50.3	145	49.7	292	100.0	
Visit to the dentist														
Yes	181	61.1	115	38.9	296	100.0	0.366*	146	49.3	150	50.7	296	100.0	0.460*
No	63	66.3	32	33.7	95	100.0		51	53.7	44	46.3	95	100.0	
Time from the last dental visit														
≤ 6 months	44	59.5	30	40.5	74	100.0	0.731*	35	47.3	39	52.7	74	100.0	0.687*
> 6 months	137	61.7	85	38.3	222	100.0		111	50.0	111	50.0	222	100.0	
Self-perception of poorly positioned teeth														
Yes	33	50.0	33	50.0	66	100.0	0.024*	16	24.2	50	75.8	66	100.0	<0.001*
No	210	64.8	114	35.2	324	100.0		181	55.9	143	44.1	324	100.0	
Type of arrangement														
Nuclear	99	62.3	60	37.7	159	100.0	0.902*	92	57.9	67	42.1	159	100.0	0.013*
Non-nuclear	144	62.9	85	37.1	229	100.0		103	45.0	126	55.0	229	100.0	
Maternal schooling														
≤ 8 years of study	109	66.9	54	33.1	163	100.0	0.167*	78	47.9	85	52.1	163	100.0	0.420*
> 8 years of study	135	60.0	90	40.0	225	100.0		117	52.0	108	48.0	225	100.0	
Monthly income														
Up to 1 minimum wage^+^	142	60.7	92	39.3	234	100.0	0.370*	124	53.0	110	47.0	234	100.0	0.408*
More than 1 minimum wage	94	65.3	50	34.7	144	100.0		70	48.6	74	51.4	144	100.0	
Social benefits^++^														
Yes	135	64.9	73	35.1	208	100.0	0.223*	103	49.5	105	50.5	208	100.0	0.754*
No	106	58.9	74	41.1	180	100.0		92	51.1	88	48.9	180	100.0	
Number of missing teeth														
None	-	-	-	-	-	-	-	191	50.8	185	49.2	376	100.0	0.412*
One or more	-	-	-	-	-	-	-	6	40.0	9	60.0	15	100.0	
Assessment of crowding in the incisal segments														
No segments crowded	-	-	-	-	-	-	-	68	48.9	71	51.1	139	100.0	0.853*
1 segment crowded	-	-	-	-	-	-	-	61	50.0	61	50.0	122	100.0	
2 segments crowded	-	-	-	-	-	-	-	68	52.3	62	47.7	130	100.0	
Assessment of spacing in the incisal segments														
No segments spaced	-	-	-	-	-	-	-	113	49.6	115	50.4	228	100.0	0.328*
1 segment spaced	-	-	-	-	-	-	-	61	55.5	49	44.5	110	100.0	
2 segments spaced	-	-	-	-	-	-	-	23	43.4	30	56.6	53	100.0	
Median diastema														
No	-	-	-	-	-	-	-	151	50.0	151	50.0	302	100.0	0.780*
Yes	-	-	-	-	-	-	-	46	51.7	43	48.3	89	100.0	
Anterior misalignment on the maxilla														
< 2 mm	-	-	-	-	-	-	-	150	50.3	148	49.7	298	100.0	0.973*
≥ 2 mm	-	-	-	-	-	-	-	47	50.5	46	49.5	93	100.0	
Anterior misalignment on the mandible														
< 2 mm	-	-	-	-	-	-	-	175	50.1	174	49.9	349	100.0	0.784*
≥ 2 mm	-	-	-	-	-	-	-	22	52.4	20	47.6	42	100.0	
Maxillary overjet														
< 4 mm	-	-	-	-	-	-	-	64	50.8	62	49.2	126	100.0	0.911*
≥ 4 mm	-	-	-	-	-	-	-	133	50.2	132	49.8	265	100.0	
Mandibular overjet														
< 4 mm	-	-	-	-	-	-	-	193	50.4	190	49.6	383	100.0	1.000^Ψ^
≥ 4 mm	-	-	-	-	-	-	-	4	50.0	4	50.0	8	100.0	
Anterior open bite														
< 2 mm	-	-	-	-	-	-	-	189	50.1	188	49.9	377	100.0	0.606*
≥ 2 mm	-	-	-	-	-	-	-	8	57.1	6	42.9	14	100.0	
Antero-posterior molar relation														
Normal	-	-	-	-	-	-	-	60	51.3	57	48.7	117	100.0	0.873*
1/2 cusp either mesial or distal	-	-	-	-	-	-	-	113	50.7	110	49.3	223	100.0	
One full cusp or more either mesial or distal	-	-	-	-	-	-	-	24	47.1	27	52.9	51	100.0	
Severe malocclusion														
Absent	-	-	-	-	-	-	-	121	49.6	123	50.4	244	100.0	0.686*
Present	-	-	-	-	-	-	-	76	51.7	71	48.3	147	100.0	
Malocclusion categories														
Normal occlusion	-	-	-	-	-	-	-	69	54.3	58	45.7	127	100.0	0.248*
Established malocclusion	-	-	-	-	-	-	-	52	44.4	65	55.6	117	100.0	
Severe malocclusion	-	-	-	-	-	-	-	40	57.1	30	42.9	70	100.0	
Very severe malocclusion	-	-	-	-	-	-	-	36	46.8	41	53.2	77	100.0	

^+^ Brazilian minimum wage in force at the time of the research equivalent to US$ 264. ^++^Bolsa Família. * Pearson’s Chi- Square test. ^Ψ^ Fisher’s Exact test. p<0.05.

In the adjusted regression analysis, the prevalence ratio (PR) of severe malocclusion was 8.4% higher among adolescents aged 12-13 years (PR = 1.08; 95% CI = 1.01-1.17) and 11.2% higher among those who perceived that their teeth were poorly positioned (PR = 1.11; 95% CI = 1.02-1.21). In turn, the negative impact on OHRQoL was approximately 74% greater in females (PR = 1.74; 95%CI = 1.40-2.15), 50% greater among those who perceived that their teeth were poorly positioned (PR = 1.50; 95% CI = 1.24-1.81) and 24.3% higher in individuals whose family arrangement was non-nuclear (PR = 1.24, 95% CI = 1.01-1.53) (Table 6).

**Table 6:** Poisson multiple regression model between dependent variables, presence of severe malocclusion and impact on adolescents’ OHRQoL, and independent variables.

Variables	Severe malocclusion				OHRQoL			
	PR Crude (CI 95%)	p-value	PR Adjusted (CI 95%)	p-valor	PR Crude (CI 95%)	p-valor	PR Adjusted (CI 95%)	p-valor
Gender								
Male	-	-	-	-	1		1	
Female	-	-	-	-	1.84 (1.48-2.29)	<0.001	1.74 (1.40-2.15)	<0.001
Age group								
12-13 years old	1.08 (1.00-1.16)	0.042	1.08 (1.01-1.17)	0.034	-	-	-	-
14-15 years old	1		1		-	-	-	-
Self-perception of poorly positioned teeth								
Yes	1.11 (1.01-1.21)	0.022	1.11 (1.02-1.21)	0.020	1.72 (1.43-2.06)	<0.001	1.50 (1.24-1.81)	<0.001
No	1		1		1		1	
Type of arrangement								
Nuclear	-	-	-	-	1		1	
Non-nuclear	-	-	-	-	1.31 (1.05-1.62)	0.016	1.24 (1.01-1.53)	0.038
Maternal schooling								
≤ 8 years of study	0.95 (0.89-1.02)	0.165			-	-	-	-
> 8 years of study	1		1		-	-	-	-

PR = Prevalence Ratio; CI = Confidence Interval; p<0.05.

## DISCUSSION

There is a growing interest among orthodontic and pediatric dentistry professionals in understanding the impact of malocclusions on the quality of life of adolescents. This desire is largely due to the recognition that experiences during adolescence play an important role in personal development. Thus, dental aesthetics can be a determining factor in the perception of one’s oral health, consequently exerting a significant influence on OHRQoL.^9^

In the present study, the choice of the Dental Aesthetic Index as an orthodontic index to assess the presence and severity of malocclusion was due to its reliability, objectivity, ease of use and ability to provide important information about the condition and treatment priorities.^17^

The results revealed that maxillary overjet ≥ 4mm, dental crowding and half-cusp molar relationship were the most frequent DAI components among adolescents, with findings similar to those described in India,^17^ ^,^ ^18^ Spain,^19^ Italy^20^ and Brazil.^21^ The variation in the prevalence of different types of malocclusion can be attributed, among other aspects, to the multifactorial etiology of this condition. In this condition, genetic, environmental, cultural and socioeconomic factors interact, resulting in complex manifestations in different contexts and demographic groups.^22^ Therefore, understanding the influence of all these causes is crucial for a comprehensive assessment of malocclusion and for the development of adequate prevention and treatment strategies.

Severe malocclusion affected an important portion of the sample, therefore becoming a relevant aspect to be considered, as the inadequate positioning of dental elements, such as in crowding and overlapping situations, can create challenges for the correct oral hygiene maintenance. This fact, in turn, increases the risk of dental caries and other oral problems, which can not only further compromise aesthetics, but also the quality of life of adolescents.^23^

Furthermore, severe malocclusion is associated with greater probability of interfering with sound production, that is, as malocclusion becomes more severe or accentuated, the probability of communication failures increases.^10^ ^,^ ^11^ Other studies have identified that patients with pronounced dental and facial deformities exhibited greater introversion and emotional instability, presenting anxiety, depression, and greater susceptibility to episodes of bullying and unsociability.^3^ ^,^ ^21^ ^,^ ^24^

The prevalence of severe malocclusion was higher among younger adolescents, that is, among those aged 12-13 years. These findings contrasted with those observed by other authors who identified greater risk of malocclusion among 15-year-old schoolchildren^19^, showing a positive correlation between malocclusion and age, with the accumulation of risk factors over time being the reason for this trend.

Although in this study no association was observed between severe malocclusion and gender, self-perception of poorly positioned teeth was more prevalent in females. Differences associated with gender can affect the way an individual perceives the presence of health problems, as well as the response to medical or dental treatments, which, in turn, can impact the different aspects of OHRQoL.^2^ ^,^ ^25^ ^-^ ^27^ Considering the psychological and social factors that lead women to show greater concern with aesthetics and dentofacial appearance, the presence of severe malocclusion or even misalignment can affect female confidence.^1^ ^,^ ^18^ ^,^ ^28^

The domains with the highest frequency of negative impact on OHRQoL were psychological discomfort, social disability and psychological disability, possibly due to the social and emotional nature of adolescence, which is characterized by a period of intense psychosocial development. At this stage, individuals seek acceptance among their peers and other individuals in their social circle and develop their personal style, seeking satisfaction at both individual and collective level.^29^ Furthermore, dental aesthetics plays a significant role in the construction of self-image and of social interactions.^21^ Given the importance of the results described here, it is essential to consider that orthodontic intervention is not limited solely and exclusively to aesthetic procedures, but represents an approach that deals with physical and psychosocial aspects, contributing to general well-being. During adolescence, the professional has the opportunity to work on preventing potential aesthetic problems, avoiding more serious complications in adulthood.

Female gender had a negative impact on adolescents’ OHRQoL, as did self-perception of poorly positioned teeth. As previously mentioned, women are more diligent than men regarding oral health, possibly due to psychological and social factors associated with cultural aesthetic standards.^1^ Therefore, dental misalignment can, in fact, affect OHRQoL. The relationship between oral health and self-esteem, especially with regard to the perception of beauty and self-image, is a factor that deserves attention. In this sense, the importance of orthodontic treatment is highlighted not only to restore functionality, but also to reinforce the emotional well-being of individuals.^18^ ^,^ ^28^

The negative impact on OHRQoL was greater in the non-nuclear family arrangement. This aspect illustrates the complex interrelationship between socioeconomic determinants and presence of severe malocclusion among adolescents. This occurs because factors such as family structure are related to the degree of knowledge about healthy lifestyle habits and, consequently, to the recognition of the need for dental care.^14^

OHRQoL was not affected by DAI components, nor by the presence of severe malocclusion or severity of malocclusion. In general, most orthodontic conditions are asymptomatic, hindering their perception.^6^ However, previous studies have identified that the most serious malocclusions are those that represent the greatest negative impact on quality of life in different domains.^8^ ^,^ ^30^ During adolescence, individuals undergo physical, psychological, emotional and personality transformations and become more concerned with facial aesthetics, since appearance gains great importance at this stage of life, so that disharmonious smile or facial deformity can have a serious impact on self-esteem.^1^ Clinicians should consider subjective measures such as quality of life during treatment planning, as it can complement the needs identified by the practitioner to perform orthodontic treatment.

In view of the above, it is necessary to overcome the barriers related to access to malocclusion treatment, especially the severe type and especially among adolescents whose families do not have the necessary financial resources to pay for treatment in private dental clinics. Therefore, it is imperative to offer and expand actions and services in the public sphere. It is also important to highlight the importance of dental surgeons to comprehensively assess the clinical conditions of patients. Recognizing that malocclusion and orthodontic characteristics not only have aesthetic implications, but also serve as indicators of the propensity for dental caries and periodontal disease is fundamental to offering the best dental care conditions.

It is important to be aware of the predictive limitations of cross-sectional studies. Due to the cross-sectional nature of the study, it was not possible to investigate the cause and effect relationships. On the other hand, this methodology can be used to assess the burden of disease or health needs of a population. As strengths of this research, the population representativeness, established through sample calculation; the performance of clinical examinations by properly calibrated researchers, and the use of a validated questionnaire should be highlighted. These aspects enable the internal and external validity of the present findings. 

## CONCLUSIONS

The presence of poorly positioned teeth was identified by the participants and the prevalence of severe malocclusion was high. The psychological discomfort, social disability and psychological disability domains had a negative impact on OHRQoL. However, there was no negative impact of severe malocclusion on OHRQoL.
